# Magnetic resonance imaging findings of intracranial tuberculoma patients in a tertiary hospital in Mogadishu, Somalia: A retrospective study

**DOI:** 10.1016/j.amsu.2022.103812

**Published:** 2022-05-21

**Authors:** Ismail Gedi Ibrahim, Ahmed Adam Osman, Mohamed Gedi Shikhow, Cihan Celik, Eren Mutlu, Mohamed sheik Hassan Qalaf, Mehmet Tahtabaşı, Yahye Garad mohamed

**Affiliations:** a Department of Radiology Mogadishu Somali Turkish Training and Research Hospital, Mogadishu, Somalia; b Department of Internal Medicine Somaville University, Mogadishu, Somalia; c Department of Neurology Mogadishu Somali Turkish Training and Research Hospital, Mogadishu, Somalia

**Keywords:** Intra-cranial tuberculoma, MRI, Somalia, Neurological disease, *M. tuberculosis*

## Abstract

**Introduction:**

Tuberculosis (TB) is a fatal disease caused by Mycobacterium tuberculosis (M.TB) with over eight million annual mortality reported worldwide attributed to the disease's direct or indirect effects. Among the most severe form of M. TB is an infection of the Central nervous system (CNS-TB). This infection is characterized by meningitis, tuberculoma, and tuberculous brain abscess. Tuberculomas are the most common variety of intracranial parenchymal tuberculosis. They occur because of conglomeration and conjugation of tubercular microgranulomas, which tend to occur at the grey-white matter junction due to the arrest of the hematogenous disseminating microbes caused by a decrease in the caliber of vessels in that region. Intracranial tuberculoma shows central hypointensity compared to grey matter, seeing this centrally on T2W images is helpful, as it is not seen in most other ring-enhancing lesions.

**Objectives:**

The purpose of this study was to evaluate the findings of the magnetic resonance imaging (MRI) scan of patients with intracranial tuberculoma using retrospective hospital records.

**Methodology:**

We conducted a retrospective data analysis of 73 patients with an age range of 3–70 years between 2018 and 2021 who were diagnosed with intracranial tuberculoma using MRI features at the Radiology Department, Somali-Turkey Recep Tayyip Erdogan Hospital. All the patients' MRI were evaluated, including conventional and contrast sequences and as well as MR diffusion.

**Results:**

This study revealed that most tuberculoma patients were female with 43 (58.9%) and 30 (41.1%) male. According to age group, the majority of patients 30 (41.1%) were 18–30 years of age. Based on the distribution of the conglomerates’ tubercles, 39 (53.4%) were located in the supratentorial region, while 24 (32.9%) were found in both the supra-tentorial and infra-tentorial regions, with 10 (13.7%) residing in the infratentorial region. Interestingly, this study also discovered that the majority of the tuberculoma patients 43 (58.9%) had multifocal lesions, with 30 (41.1%) having single lesions. Also, associated abnormalities were detected in 28 (38.4%) of the patients with meningitis, while 7 (9.6%) had both hydrocephalus and meningitis, 2 (2.7%) had hydrocephalus, and one patient had cerebral infarction.

**Conclusion:**

The outcome of this investigation shows MRI as a suitable diagnostic tool for the diagnosis of intracranial tuberculoma and associated abnormalities in geographic areas where tuberculosis is endemic.

## Highlights

• Tuberculomas are the most common variety of intracranial parenchymal tuberculosis.
• MRI findings are suitable diagnostic tools for intracranial tuberculoma and associated abnormalities in patients having space-occupying lesions where tuberculosis is endemic.
• Early diagnosis and management will reduce the likelihood of irreversible neurological consequences and unnecessary biopsies for the patient.

## 1 Introduction

Tuberculosis (TB) is still a significant infectious disease, with 10.4 million new cases and 1.8 million deaths worldwide in 2016. In 2019, approximately 10.0 million tuberculosis (TB) cases were diagnosed worldwide [1,2]. In 2014, the average TB incidence in Somalia was 274 cases per 100,000 people, with a prevalence of 513 cases per 100,000 people [3]. Neurotuberculosis is a type of extrapulmonary tuberculosis with a high mortality and morbidity rate [1]. Tuberculomas are the most common variety of intracranial parenchymal tuberculosis. Tuberculomas can present as solitary or multiple tuberculomas and can also occur with or without meningitis [4]. The cornerstone of CNS TB diagnosis and associated problems is imaging. The modality of choice is commonly contrast-enhanced magnetic resonance imaging (MRI). CNS TB's clinical and radiologic symptoms might be mistaken for other infectious and non-infectious neurological disorders such as brain tumors and non-TB-related granulomas like sarcoid. A greater understanding of the imaging features of CNS TB will help with a clear and precise diagnosis [5,6].

The purpose of this study was to describe the magnetic resonance imaging results of intracranial tuberculomas that can be misdiagnosed as space-occupying lesions.

## 2 Methodology

We conducted a retrospective data analysis of 73 patients with an age range of 3–70 years between 2018 and 2021 who were diagnosed with intracranial tuberculoma using MRI features at the Radiology Department, Somali-Turkey Recep Tayyip Erdogan Hospital.

Diagnosis of tuberculoma was confirmed by typical MRI presentation with some patients with chest TB infiltration and some patients with positive acid-fast bacilli tests were included. All the patients' cranial magnetic resonance imaging (MRI) was evaluated, including conventional T1-weighted [409/11 (TR/TE)] and T2-weighted [3300/102 (TR/TE)] images as well as contrast-enhanced T1 and diffusion-weighted images using the MRI 1.5-T scanner. The number of tuberculoma lesions, their locations, size, and signal characteristics were all recorded. According to signal characteristics, the tuberculoma lesions are divided into caseating and noncaseating granulomas.

Patients with non-specific tuberculoma MRI findings and clinical presentation were excluded from the study.

The data was collected using Microsoft excel and was analyzed using Statistical Package for Social Sciences (IBM SPSS) software version 26. Descriptive data analysis.

Research approved by the ethical committee in Mogadishu Somali Turkey, education and Research Hospital 24-1-2022 decision NO. 468 MSTH/8878. The study followed the criteria of the STROCSS guideline [20].

## 3 Results

**Fig. 1:**
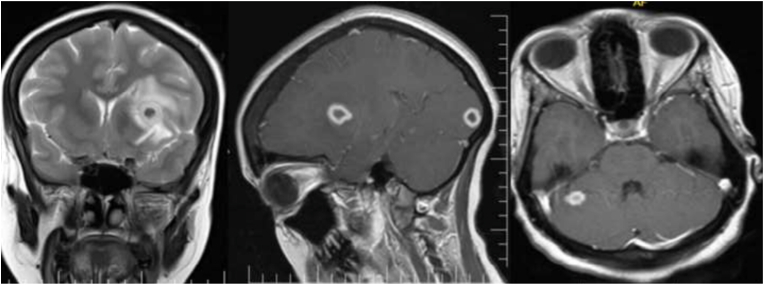
Coronal T2 (A) appears hypointense and post-contrast axial (B) and sagittal (C) images show multifocal ring enhancement in supratentorial and infratentorial conglomerate tuberculomas.

**Fig. 2:**
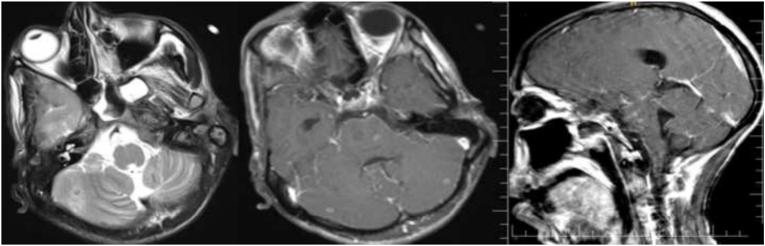
Axial T2 (A) appears hypointense and post-contrast axial (B) and sagittal (C) images show multiple enhancement infratentorial conglomerate tuberculomas.

**Fig. 3:**
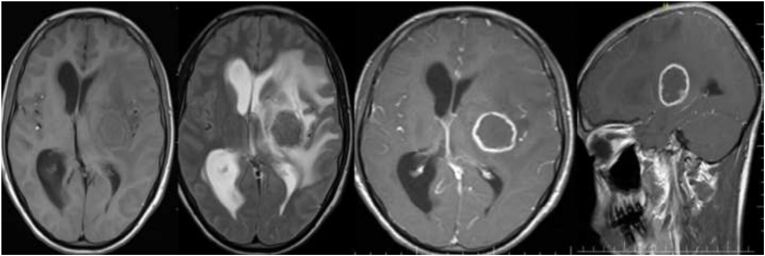
caseating tuberculomas A (T1W axial) appears isointense with a hyperintense rim. B (FLAIR axial) hypointense lesions in the left parietal lobe with perilesional edema, which in post-contrast C (T1W axial) and D (T1W sagittal) show ring enhancement of the conglomerate tuberculomas.

**Fig. 4:**
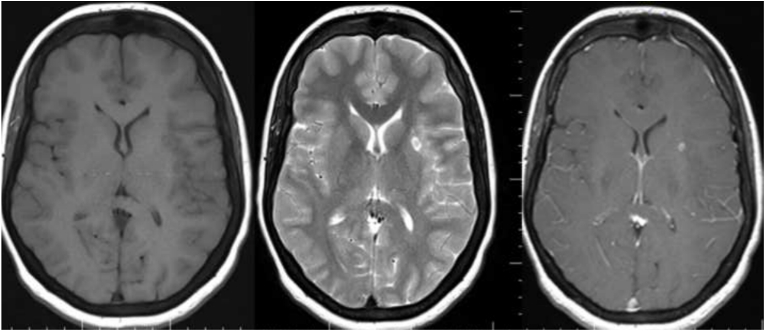
Noncaseating granuloma. (A) Tuberculoma in the left basal ganglia is hypointense in the T1-weighted axial image, (B) hyperintense in the T2-weighted axial image. (C) Nodular contrast enhancement of the tuberculoma on the post-contrast image.

**Fig. 5:**
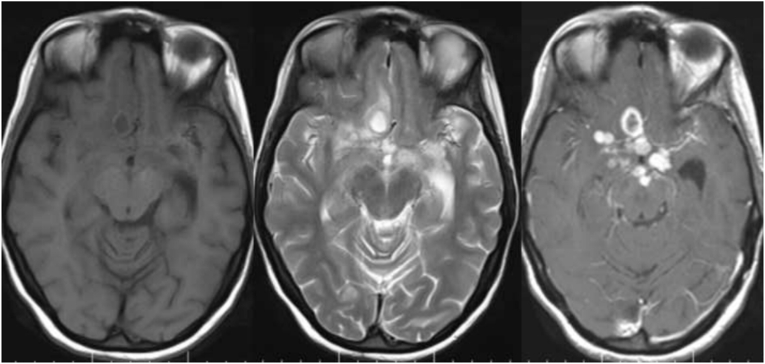
noncaseating granuloma. (A) tuberculoma in the frontal and suprasellar regions, hypointense in the T1-weighted axial image, (B) hyperintense in the T2-weighted axial image, and (C) ring contrast enhancement of the tuberculoma on the post-contrast image.

**Fig. 6:**
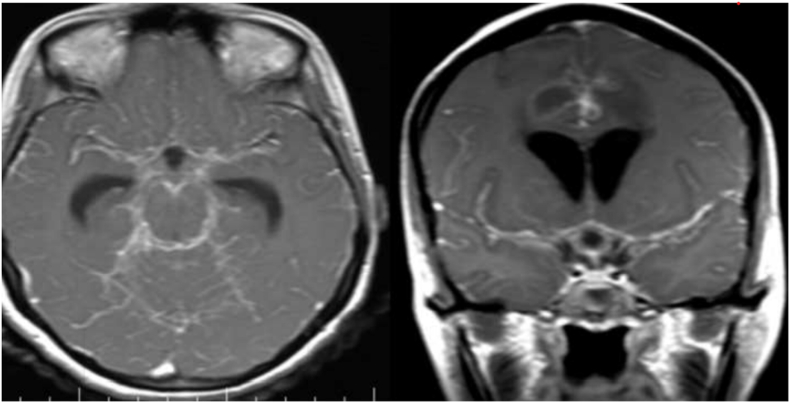
Tuberculous leptomeningitis in the post-contrast images A (T1 axial) and B (T1 coronal) show diffuse leptomeningeal enhancement with the basal cisterns, Sylvian fissures, and cerebellar folia.

The findings of this study revealed that the majority of tuberculoma patients were female, with 43 (58.9%), and male wıth 30 (41.1%). According to age group, the majority of patients 30 (41.1%) were 18–30 years of age. Based on the distribution of the conglomerates’ tubercles, 39 (53.4%) were located in the supratentorial region(Fig. 3), while 24 (32.9%) were found in both the supra-tentorial and infra-tentorial regions(Fig. 1), with 10 (13.7%) residing in the infratentorial region(Fig. 2). This study also discovered that the majority of the tuberculoma patients, 43 (58.9%) had multifocal lesions, with 30 (41.1%) having single lesions. Tuberculoma size varied between <2.5 and >2.5 cm. 44 (60.3%) of the lesions were smaller than 2.5 cm, and 29 (39.7%) were greater than 2.5 cm. Tuberculoma lesions were classified as caseating or noncaseating granulomas depending on their signal intensities and contrast enhancement characteristics. According to our findings, 67 (91.8%) were caseating(Fig. 3) and 6 (8.2%) were noncaseating granulomas (Fig. 4, Fig. 5). According to features of edema, this study found that in most the tuberculoma patients, 52 (71.2%) have edema and mass effect, and 21 (28.8%) have no edema and mass effect. Also, associated abnormalities were detected in 28 (38.4%) of the patients with meningitis(Fig. 6), while 7 (9.6%) had both hydrocephalus and meningitis, 2 (2.7%) had hydrocephalus, and one patient had cerebral infarction (Table 1).

**Table 1:** Features of tuberculoma and associated abnormalities.

		Number	percentages
Number of lesions	Single lesion	30	41.1%
	Multifocal lesion	43	58.9%
Location of lesion	Supratentorial	39	53.4%
	Infratentorial	10	13.7%
	Both	24	32.9%
Size of the lesion	<2.5 cm	44	60.3%
	>2.5 cm	29	39.7%
Type of tuberculoma	Caseating	67	91.8%
	Non-caseating	6	8.2%
Features of edema and mass effect	edema and mass effect	52	71.2%
	No edema and mass effect	21	28.8%
Associated abnormality	No abnormality	35	47.9%
	Hydrocephalus	2	2.7%
	Meningitis	28	38.4%
	Hydrocephalus and meningitis	7	9.6%
	Infraction	1	1.4%

## 4 Discussion

Tuberculosis (TB) is still a major global problem because of the increasing incidence of the human immunodeficiency virus (HIV) and drug-resistant strains, although its incidence has recently declined [7].

In both developing and developed countries, tuberculosis (TB) has been a major public health issue. It is, nevertheless, a disease surrounded by poverty, economic suffering, susceptibility, and marginalization, as well as stigma and discrimination among those who are affected [8]. Among the most severe form of M. TB is an infection of the Central nervous system (CNS-TB). This infection is characterized by meningitis, tuberculoma, and tuberculous brain abscess [9]. Tuberculosis that affects the central nervous system (CNS) accounts for 10%–15% of all tuberculous illnesses [10].

Intracranial tuberculomas are granulomatous tissue masses that develop as a result of hematogenous dissemination from a distant tuberculous infection [11]. They occur because of the conglomeration and conjugation of tubercular microgranulomas that tend to occur at the grey-white matter junction due to arrest of the hematogenous disseminating microbes caused by a decrease in the caliber of vessels in that region [9].

Different forms of tuberculoma are characterized based on MRI findings [4]. On T1-and T2-weighted scans, caseating solid granulomas are frequently hypointense and markedly hypointense, respectively. The central core's granulation tissue and compressed glial tissue result in a higher cellular density than the brain parenchyma, resulting in relative hypointensity. Noncaseating granulomas are frequently hypointense to isointense on T1-weighted scans and hyperintense on T2-weighted images, with no distinctive imaging features. Following the application of contrast media, homogeneous enhancement can be detected [12]. Sonmez et al. [13] reported that there were 85% caseating tuberculoma granulomas., and those noncaseating granulomas were 14.1%. Similarly to our findings 91.8% were caseating and 8.2% were noncaseating granulomas.

Tuberculomas can occur anywhere in the brain, but are most common in the frontoparietal region and basal ganglions, and very rarely in the corpus callosum, quadrigeminal cistern, pontocerebellar angle, and retro-orbital regions [14]. Azeemuddin et al. [5]reported that supratentorial tuberculomas have 64% and infratentorial locations have 36%. Sonmez et al. [13] reported that tuberculoma involvement in the cerebral hemisphere was 64%, the cerebellar hemisphere was 26.5%, and that of the brain stem was 9.5%. Similarly, our study showed that 53.4% of the lesions were located in the supratentorial region, while 24 (32.9%) were found in both the supra-tentorial and infra-tentorial regions. In 10%–34% of cases, there may be multiple lesions [6,15]. Sonmez et al. [13]reported that the lesions were multiple in 89% of cases. İn other hand our study, 58.9% had multifocal lesions, and 41.1% had single lesions.

Tuberculomas lesion size varied in less than 2.5 cm and more than 2.5 cm. Gupta et al. [16] found less than 2.5 cm in size of the lesions, which was 88%. Sonmez et al. [13]observed less than 2.5 cm of the lesion at 90.5%. In our study 60.3% of the lesions were smaller than 2.5 cm in size.

Tuberculous meningitis (TBM) is the most frequent manifestation of neurotuberculosis, affecting mostly young children and adolescents [17]. In patients with tuberculous meningitis, hydrocephalus can be communicating, noncommunicating, or complex. Tubercular hydrocephalus, which accounts for 80% of cases, is usually communicative. Inflammatory exudate obstructs CSF flow in the basal cisterns, resulting in this condition. In other situations, the hydrocephalus is non-communicating due to tuberculomas or, much less commonly, tuberculous abscesses [7,18]. Kilani et al. [19] found a combination of meningitis and tuberculoma at a level of 9.8%. Sonmez et al. [13] reported that hydrocephalus was 18.5%. In contrast our study showed that 38.4% of the patients have meningitis 9.6% had both hydrocephalus and meningitis, 2.7% had only hydrocephalus.

Sonmez et al. reported in their study that the edema and mass effects in the surrounding tissues were present in 4% of the cases. In contrast to our study, we found edema and mass effects in most of the tuberculoma patients 71.2%.

## 5 Conclusion

The outcome of this research showed that MRI findings are suitable for the diagnosis of intracranial tuberculoma and associated abnormalities in patients having space-occupying lesions where tuberculosis is endemic, and early diagnosis and management will reduce the likelihood of irreversible neurological consequences and unnecessary biopsies for the patient.

## Sources of funding for your research

There are no sponsors or funding sources for this work.

## Ethical approval

Research approved by the ethical committee in Mogadishu Somali Turkey, education and Research Hospital 24-1-2022 decision NO. 468 MSTH/8878.

## Consent

Authors have taken written consent from the patient's mother, and it will be available on request.

## Author contribution

Ismail Gedi Ibrahim; written literature review, introduction, and parts of the discussion. Mohamed Gedi Shikhow: Data analysis Ahmed Adam Osman: Interpretation. Cihan CELIK^:^ Abstract writing. Eren MUTLU: Radiological diagnosis Mohamed sheik Hassan (Qalaf): reviewed patients' data and some part of the discussion. Mehmet TAHTABASI: scientifically reviewed. Yahye Garad Mohamed: reviewed patients’ data.

## Registration of research studies

1. Name of the registry: Not applicable
2. Unique Identifying number or registration ID: Not applicable
3. Hyperlink to your specific registration (must be publicly accessible and will be checked):

## Guarantor

Ismail Gedi Ibrahim.

## Declaration of competing interest

Authors have no financial or personal conflict that can influence this work.

## References

[1] Priya B., Govindarajan T.G. (2017). Dilemmas in the diagnosis and treatment of intracranial tuberculomas. J. Neurol. Sci..

[2] Chakaya J., Khan M., Ntoumi F., Aklillu E., Fatima R., Mwaba P. (2021).

[3] Ali M.K., Karanja S., Karama M. (2017). Factors associated with tuberculosis treatment outcomes among tuberculosis patients attending tuberculosis treatment centres in 2016-2017 in Mogadishu, Somalia. Pan Afr Med J.

[4] Sadashiva N., Tiwari S., Shukla D., Bhat D., Saini J., Somanna S. (2017). Isolated brainstem tuberculomas. Acta Neurochir..

[5] Azeemuddin M., Alvi A., Sayani R., Khan M.K., Farooq S., Beg M.A. (2019). Neuroimaging findings in tuberculosis: a single-center experience in 559 cases. J. Neuroimaging.

[6] Bargallo N., Berenguer J., Tomas X., Nicolau C., Cardenal C., Mercader J.M. (1993). Intracranial tuberculoma: CT and MRI. Eur. Radiol..

[7] Patkar D., Narang J. (2012). Central Nervous System Tub e rc u l o s i s Pathophysiology and Imaging Findings. Neuroimaging Clin NA.

[8] Perez-malagon C.D., Barrera-rodriguez R., Lopez-gonzalez M.A., Alva-lopez L.F. (2021). Diagnostic and neurological overview of brain tuberculomas : a review of literature.

[9] Khatri G.D., Krishnan V., Antil N., Saigal G. (2018). Magnetic resonance imaging spectrum of intracranial tubercular lesions: one disease, many faces. Pol. J. Radiol..

[10] Li H., Liu W., You C. (2012). Central nervous system tuberculoma. J. Clin. Neurosci..

[11] Salgado P., Del Brutto O.H., Talamas O., Zenteno M.A., Rodríguez-Carbajal J. (1989). Intracranial tuberculoma: MR imaging. Neuroradiology.

[12] Taheri M.S., Karimi M.A., Haghighatkhah H., Pourghorban R., Samadian M., Kasmaei H.D. (2015).

[13] Sonmez G., Ozturk E., Sildiroglu H.O., Mutlu H., Cuce F., Senol M.G. (2008). MRI findings of intracranial tuberculomas. Clin. Imag..

[14] Oncul O., Baylan O., Mutlu H., Cavuslu S., Doganci L. (2005). Tuberculous meningitis with multiple intracranial tuberculomas mimicking neurocysticercosis clinical and radiological findings. Jpn. J. Infect. Dis..

[15] Guzel A., Tatli M., Aluclu U., Yalcin K. (2005). Intracranial multiple tuberculomas: 2 Unusual cases. Surg. Neurol..

[16] Gupta R.K., Jena A., Singh A.K., Sharma A., Puri V., Gupta M. (1990). Role of magnetic resonance (MR) in the diagnosis and management of intracranial tuberculomas. Clin. Radiol..

[17] Bernaerts A., Vanhoenacker F.M., Parizel P.M., Van Goethem J.W.M., van Altena R., Laridon A. (2003). Tuberculosis of the central nervous system: overview of neuroradiological findings. Eur. Radiol..

[18] Bathla G., Khandelwal G., Maller V.G., Gupta A. (2011). Manifestations of cerebral tuberculosis. Singap. Med. J..

[19] Kilani B., Ammari L., Tiouiri H., Goubontini A., Kanoun F., Zouiten F. (2003). Manifestations neuroradiologiques initiales de la tuberculose du système nerveux central de l’adulte. À propos de 122 cas. Rev. Med. Interne.

[20] Mathew G., Agha R., Albrecht J., Goel P., Mukherjee I., Pai P., D'Cruz A.K., Nixon I.J., Roberto K., Enam S.A., Basu S. (2021 Dec 1). Strocss 2021: strengthening the reporting of cohort, cross-sectional and case-control studies in surgery. Int. J. Surg. Open.

